# Hydrate Chloral

**Published:** 1874-12

**Authors:** Alban S. Payne

**Affiliations:** Virginia


VOL. IV.—DECEMBER, 1874.—No. 12.

### Original Communications.

### HYDRATE CHLORAL.

BY ALBAN S. PAYNE, M.D., OF VIRGINIA.

This remedial agent has never received from the great mass of the profession that attention and consideration which its preëminent virtues entitle it to. With a great many physicians there appears to be a disinclination—a reluctance—to administer chloral. Since the spring of 1870 I have been in the habit of prescribing the hydrate of chloral in most diseases where there was a perceptible derangement of the nervous system, whether arising from a prolonged source of morbific agents, or from a sudden "shock" to the nervous system; and after a somewhat extensive experience, and a careful analysis of its therapeutical action, I have formed a very high opinion of its surprising value as a remedial agent. Indeed, I am disposed to believe, as an internal remedy, the discovery of this drug to be the greatest boon to suffering humanity that has been discovered for the last half of a century. I have some old, long-standing cases of "*mania a potu*" that I have been for a number of years occasionally called upon to treat, which have afforded me a fine opportunity to test the relative value between the old plan of treatment (with the accustomed stimulus at regular intervals), and the more recent plan by the administration of chloral. And I find this to be about the difference: whilst by the old plan, if the patient was enabled to attend to his business in four to six days, I congratulated myself and the patient; with the hydrate chloral, thirty grains at four or five o'clock P.M., and sixty grains at bed-time, they rarely fail to enjoy a comfortable night's sleep, and in the morning they awake refreshed, nerves braced, and go about their occupations as if nothing unusual had occurred to them.

The beneficial effects of this remedy in delirium tremens is *simply wonderful.* No prostration appears to follow the stimulating effects of chloral, as it does in all other drugs; nor is there the same "craving" for alcoholic stimulants after chloral that there always is after the administration of opiates and the other usual stimulants. Another remarkable difference between hydrate chloral and all other drugs is, that it is somewhat beneficial in any sized dose, although given greatly under its normal dose. In other words, it makes a patient feel cheerful, comfortable, although the dose given is not potent enough to put him to sleep. With opiates and common stimulants this is never the case; you must give your patient enough to produce the desired effect, the specific effect of the drug, or you do your patient positive harm rather than benefit him by their exhibition; you excite, rather than compose him. A fifteen-grain dose of chloral will benefit your patient somewhat, when at the same time it will require sixty grains to enable him to sleep. It seems to act as a sort of "pabulum" for the nerves. I have repeatedly given chloral in as small a dose as fifteen grains, and in as large a dose as one hundred grains, without ever seeing a single unpleasant symptom therefrom. I prescribed chloral the past summer in two cases of "*coup de soliel,*" with the happiest effect. Cold water to the surface of the body and head, and chloral internally, I think are the *reliable* remedies in sun-stroke. It does not arrest or check any of Nature's secretions.

It is rather a moderate promoter of all the secretions, for I have found in cases where there is a paucity of high-colored urine, it sensibly increases the flow, and gives manifest tone and power to the bladder. When the bowels are overloaded, yet constipated, it acts as a mild, safe aperient. I have no doubt that in diarrhœas and dysenteries caused by atony and irritability of the bowels, chloral hydrate would prove itself almost a "specific." It is a moderate promoter of digestion, and rarely fails to increase the appetite. In many cases of dyspepsia, tincture of lupulin, combined with small doses of chloral, prove themselves of signal value. Especially is this the case in the dyspepsias of the old debauché. Food taken after the administration of chloral does not oppress the stomach as it does after the administration of the common stimulants and appetizers. As its only sensible effect is to rouse, *brace* the nervous system, it interferes unfavorably with no other remedy, but rather assists in their therapeutic effect upon the system. I have repeatedly prescribed the hydrate in protracted cases of labor, from rigidity of the perineal muscles or os uteri, with as beneficial results as I ever gained from venesection along with the regular, persistent use of the antimonii et potassa tartras. Seeing the apparent dread of some able practitioners of medicine to administer chloral, induced me to experiment with this drug quite extensively in my own person. I have often taken as little as five grains, and as often one hundred grains, and always with a pleasant effect—a remarkably pleasant effect. I am aware that many able medical men caution you in its use, for fear of producing death by paralysis of the pneumogastric nerve. But then a half century ago it was the universal opinion of the best medical men that a drink of cold water upon calomel was necessarily fatal. A more thorough experience has proven the administration of calomel with a plenty of ice and ice-water to be the preferable plan. So I think it will prove with chloral. This fear of producing a paralysis of the pneumogastric nerve is more fanciful than real. I hardly think an over-dose of chloral more likely to produce paralysis of the par-vagum and its branches than an over-dose of brandy. We all know if a man goes to a graveyard expecting to see a ghost, he is very apt to see one. Chloral is supposed to be the cause from manner of death. The doctor administers chloral; he fears paralysis of the pneumogastric nerve; the patient dies from the "want of breath;" chloral is believed to have produced the fatal result, when it is only chargeable with having failed to relieve the patient, and nothing more. All deaths are directly or remotely from want of breath. For instance, a man is thrown from his horse, in full view; you reach him, he is dead; examine him and find dislocation and fracture of one or two of the cervical vertebræ. A friend asks you what was the cause of death; you tell him his horse threw him and broke his neck. This is not correct, for here, too, the patient evidently died from the want of breath. The fact is, the fall fracturing the cervical vertebræ, drove them inwards and downwards, thereby producing pressure upon the eighth pair of nerves, composed of the glosso-pharyngeal, pneumogastric and spinal accessory; the lungs, thus deprived of their accustomed supply of nervous stimulus, collapse, and the patient dies here, too, for the "want of breath." Should the fractured vertebræ be driven from, not down upon, these important nerves, there is nothing in this accident incompatible with life and even health. I have seen in my life—once in the human subject and twice in the horse—literally speaking a "broken neck," yet the patients in each case were in the enjoyment of sound, vigorous health. I do not believe it has so much specific power over the par-vagum and the anterior pulmonary plexus as it is given credit for having. Hence I have been somewhat disappointed in the hydrate as a remedy in spasmodic asthma. If asthma is not sympathetic, and dependent upon the condition and contents of the stomach from some indigestible substance, which is best relieved by active bilious purgatives of calomel and jalap, I think nitric acid, large doses of iodide of potassium, and arsenic, will be found to be the best remedies.

